# Hyperpolarized *in vivo* pH imaging reveals grade-dependent acidification in prostate cancer

**DOI:** 10.18632/oncotarget.27225

**Published:** 2019-10-22

**Authors:** David E. Korenchan, Robert Bok, Renuka Sriram, Kristina Liu, Romelyn Delos Santos, Hecong Qin, Iryna Lobach, Natalie Korn, David M. Wilson, John Kurhanewicz, Robert R. Flavell

**Affiliations:** ^1^ Department of Radiology and Biomedical Imaging, University of California, San Francisco, CA, USA; ^2^ Department of Epidemiology and Biostatistics, University of California, San Francisco, CA, USA; ^3^ Department of Pharmaceutical Chemistry, University of California, San Francisco, CA, USA; ^4^ Department of Urology, University of California, San Francisco, CA, USA; ^5^ Department of Physical Chemistry, Technical University of Munich, Munich, Germany

**Keywords:** prostate cancer, extracellular pH, hyperpolarization, MRI, metabolism

## Abstract

There is an unmet clinical need for new and robust imaging biomarkers to distinguish indolent from aggressive prostate cancer. Hallmarks of aggressive tumors such as a decrease in extracellular pH (pH_e_) can potentially be used to identify aggressive phenotypes. In this study, we employ an optimized, high signal-to-noise ratio hyperpolarized (HP) ^13^C pH_e_ imaging method to discriminate between indolent and aggressive disease in a murine model of prostate cancer. Transgenic adenocarcinoma of the mouse prostate (TRAMP) mice underwent a multiparametric MR imaging exam, including HP [^13^C] bicarbonate MRI for pH_e_, with ^1^H apparent diffusion coefficient (ADC) mapping and HP [1-^13^C] pyruvate MRI to study lactate metabolism. Tumor tissue was excised for histological staining and qRT-PCR to quantify mRNA expression for relevant glycolytic enzymes and transporters. We observed good separation in pH_e_ between low- and high-grade tumor regions, with high-grade tumors demonstrating a lower pH_e_. The pH_e_ also correlated strongly with monocarboxylate transporter *Mct4* gene expression across all tumors, suggesting that lactate export via MCT4 is associated with acidification in this model. Our results implicate extracellular acidification as an indicator of indolent-to-aggressive transition in prostate cancer and suggest feasibility of HP pH_e_ imaging to detect high-grade, clinically significant disease in men as part of a multiparametric MRI examination.

## INTRODUCTION

Current clinical management of prostate cancer is lacking in the ability to confidently distinguish indolent from aggressive tumors. As of 2019, prostate cancer had the highest incidence rate, 20% of all new cases, and the second-highest mortality rate, responsible for 10% of all cancer deaths [1]. The majority of prostate cancer diagnoses, however, are tumors that do not require treatment [2]. For patients with prostate tumors that are likely indolent, typically having less than 0.5 cc of volume and a Gleason grade below 3+3, active surveillance represents a clinical standard for detecting indolent-to-aggressive transition [3]. Imaging, and in particular multiparametric ^1^H MRI, plays a prominent role in active surveillance because of its ability to localize aggressive focal lesions within the prostate for focal therapy [4]. Nevertheless, prostate MRI still suffers from a high incidence of both false positive [5] and false negative [6] events, requiring other biomarkers and associated imaging techniques for delineation of focal lesions.

Prostate cancer demonstrates prominent metabolic alterations from native tissue that may be detectable via molecular imaging. The glycolytic shift of prostate cancer cells from citrate secretion to lactate production, measurable in biological samples or in living systems using magnetic resonance (MR) techniques [7, 8], is one parameter that has demonstrated considerable potential for indicating cancer aggressiveness. Increased production of lactate can be measured with hyperpolarized (HP) ^13^C MR, which relies upon dramatic MR signal enhancement provided by dynamic nuclear polarization (DNP) [9]. This technique has been translated into clinical research studies, and the increased production of lactate can be directly visualized through imaging following administration of [1-^13^C] pyruvate [10]. Furthermore, multiple biological parameters can be simultaneously measured in the same imaging session with this technique [11]. Other cellular changes associated with this metabolic shift have also been identified. In particular, increased lactic acid export via monocarboxylate transporter 4 (MCT4) overexpression has been observed in high-grade renal cell carcinoma cell lines [12] and patient-derived tissue slices [13]. The MCT4 transporter is overexpressed in aggressive murine prostate cancer [14] and patient-derived tissue slices [15]. Although this increase in lactic acid efflux can be measured *in vivo* using diffusion-weighted imaging [16], the inherent changes in cellularity with tumor grade in prostate cancer obfuscate the changes in the apparent diffusion coefficient and reduce the dynamic range between intracellular and extracellular compartments.

Extracellular acidification, in part a consequence of this altered metabolism, represents a possible biomarker for the detection of aggressive prostate cancer [17]. Solid tumors typically have high metabolic activity and rapid cell proliferation; consequently, they develop an acidic interstitial microenvironment (pH 6.5–7.2). Acidic extracellular pH is associated with local invasion and metastasis in a variety of cancers, including melanoma, breast and colon cancer [18–21]. Evaluation of prostate cancer models has demonstrated that these tumors have an acidic extracellular pH [11, 22]. Moreover, treatment with pH-increasing therapies such as sodium bicarbonate inhibits tumor formation in prostate cancer models [23]. A variety of methods have been developed for imaging pH *in vivo* [24], including hyperpolarized ^13^C magnetic resonance techniques [22, 25–28]. Recently, we have developed a method for high signal-to-noise ratio (SNR) imaging of extracellular pH using a precursor molecule, [1-^13^C] glycerol carbonate, which can be converted to [^13^C] bicarbonate, administered to an animal, and rapidly imaged to generate maps of tumor pH_e_ [22]. Importantly, this method is anticipated to have a low barrier to entry into the clinic, considering the low toxicity of sodium bicarbonate.

We hypothesized that alterations in tumor pH_e_, metabolism, and cellularity could represent imaging biomarkers of low- to high-grade cancer transition in prostate cancer. In order to test this hypothesis, we applied our recently-developed HP pH_e_ imaging method, in combination with [1-^13^C] pyruvate imaging and ^1^H diffusion-weighted imaging, in a cohort of transgenic adenocarcinoma of the mouse prostate (TRAMP) mice [29]. The imaging findings were compared with pathologic outcome and enzyme/transporter gene expression measurements.

## RESULTS

### ^1^H MR imaging and mouse model

**Figure 1:**
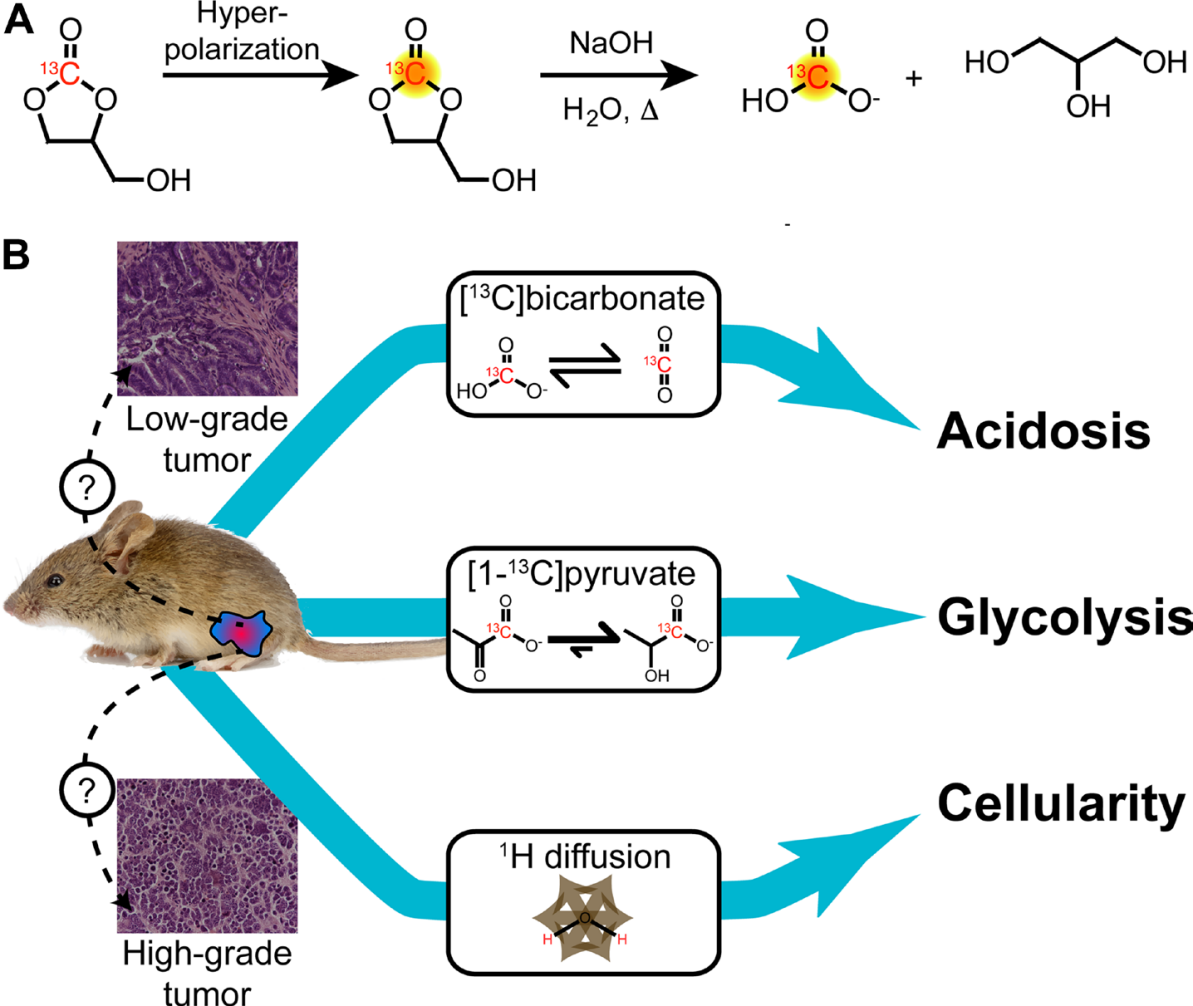
Schematic of multiparametric MR imaging protocol utilized in this work. (**A**) Hyperpolarized [^13^C] bicarbonate was obtained by polarizing [1-^13^C]1,2-glycerol carbonate, which was rapidly hydrolyzed immediately prior to injection using base and heat, followed by neutralization. This approach generates a pH-neutral solution of HP [^13^C] bicarbonate with high signal for *in vivo* pH_e_ imaging. (**B**) Transgenic mice with prostate lesions were subjected to ^1^H diffusion-weighted imaging as well as two separate hyperpolarized ^13^C injections of [1-^13^C] pyruvate and [^13^C] bicarbonate with MR spectroscopic imaging in a single imaging study. These imaging methods enabled measurement of tumor cellularity, glycolytic metabolism, and extracellular acidification, respectively.

We developed and implemented a new multiparametric MR protocol to study extracellular acidification, glycolytic metabolism, and tumor cellularity in a single imaging exam (Figure 1). Hyperpolarization and rapid hydrolysis of [1-^13^C]1,2-glycerol carbonate generated a solution of highly polarized and concentrated [^13^C] bicarbonate along with an equimolar concentration of glycerol, as previously reported by our group [22]. Notably, the percent polarization and concentration of the [^13^C] bicarbonate (19% and 100 mM) was similar to that of [1-^13^C] pyruvate (18% and 80 mM), when back-calculated to the time of dissolution. The [^13^C] bicarbonate signal enabled pH_e_ imaging in mouse prostate tumors with sufficient spatial resolution to capture tumor heterogeneity. This was combined with imaging of hyperpolarized [1-^13^C] lactate produced from [1-^13^C] pyruvate and ^1^H diffusion-weighted imaging in order to study glycolysis, acidosis, and tumor cellularity in a single imaging exam.

**Figure 2:**
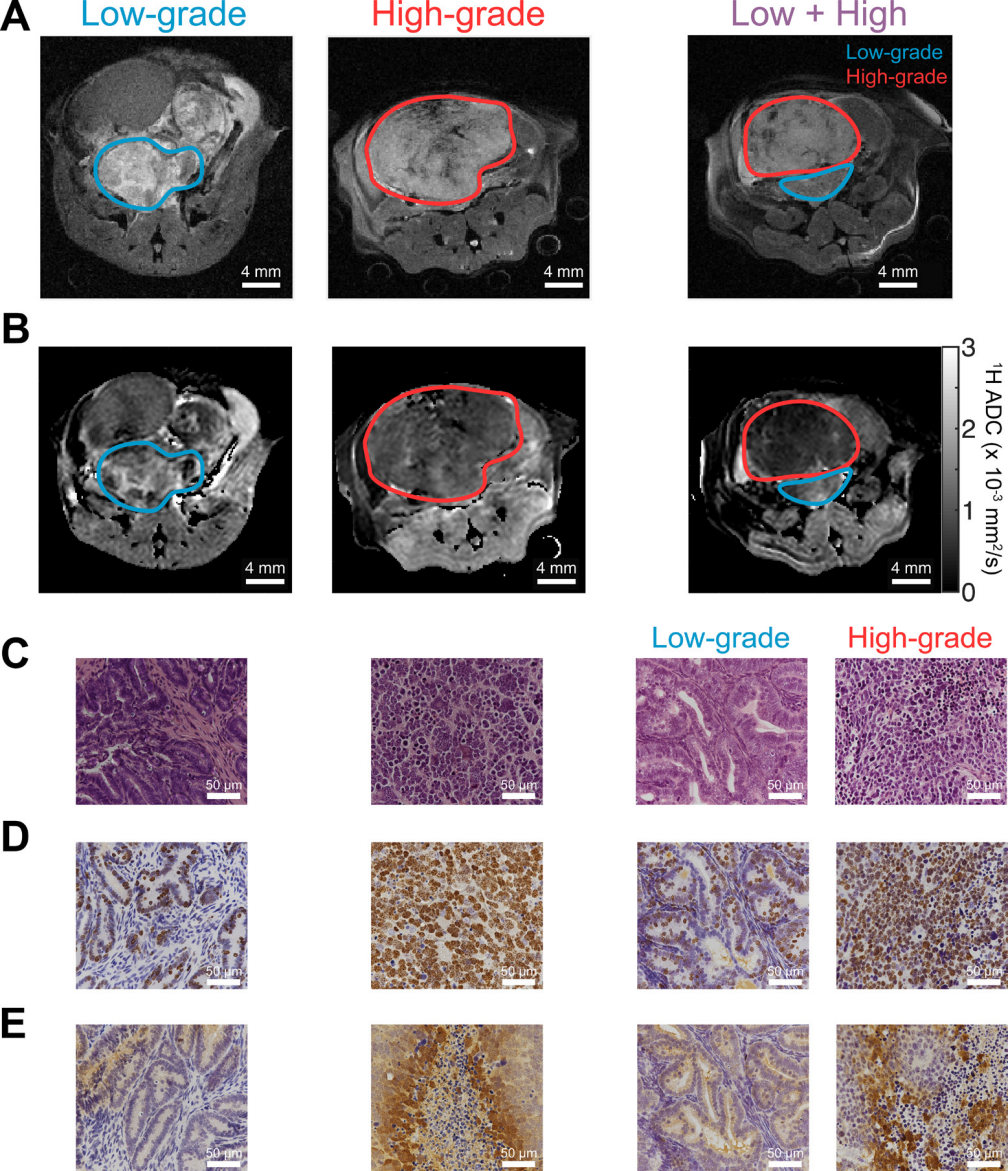
^1^H MR imaging and histological staining of TRAMP tumor tissue display differences in aggressive phenotype. (**A**) Anatomical ^1^H MR images of TRAMP tumors during different stages of tumor progression, displaying predominantly low-grade lesions, predominantly high-grade lesions, and lesions with distinct low- and high-grade regions (“Low + High”), identified by MRI and confirmed via histology. These correspond with mice 2, 9, and 5, respectively, as listed in the Supporting Information and Methods, Supplementary Table 1. (**B**) ^1^H apparent diffusion coefficient (ADC) maps for the same mice as in part (A), demonstrating a reduction in ADC in high grade tumors. (**C**–**E**) Tumor tissue corresponding with MRIs in parts (A–B) and stained with (C) H&E (D) Ki-67 proliferation marker stain; and (E) anti-pimonidazole hypoxia stain. Images demonstrate differences in morphology, cellularity, and hypoxia between low- and high-grade tumor tissues. All microscope images were acquired with 40× magnification.

We chose to use the genetically engineered TRAMP mouse model in order to study metabolic, morphologic, and microenvironmental changes. This mouse model is known to recapitulate the phenotype of human prostate cancer, particularly with regards to lactate metabolism [8, 14, 30], and progresses through low- and high-grade stages analogous to human prostate cancer [31]. We performed HP and conventional MR imaging on a total of 10 TRAMP mice. Anatomical ^1^H images as well as ^1^H apparent diffusion coefficient (ADC) maps revealed considerable inter- and intra-tumoral heterogeneity (Figure 2A, 2B). Eight of the 10 mice displayed lesions that could be grossly classified histologically as either “low-grade,” characterized by well- or moderately-differentiated cells, largely-preserved glandular structure, and normal or above-normal cellularity, or “high-grade,” highly cellular lesions containing sheets of pleomorphic, poorly-differentiated cells (Figure 2C). This classification was performed in a similar manner as with human prostate clinical biopsies, as previously described [8, 30]. Supplementary Table 1 summarizes the composition of tumor based upon differentiation. Interestingly, 2 of the 10 mice contained distinctly separate regions of low- and high-grade cancer, as determined by histology, which also demonstrated differences in contrast on ^1^H anatomical images (Figure 2, 3rd and 4th columns). These findings recapitulate features of human prostate cancer, where mixed tumors including both low- and high-grade components are common.

**Figure 3:**
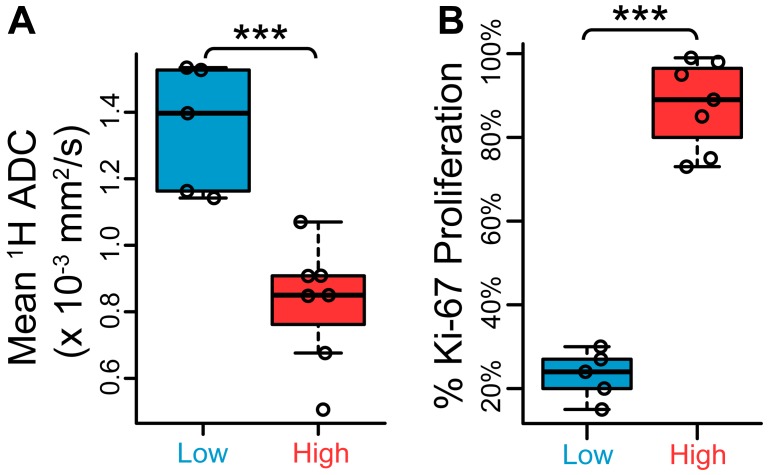
Low- and high-grade TRAMP lesions exhibit differences in ^1^H apparent diffusion coefficient (ADC) and Ki-67 proliferation staining. (**A**) Scatter plots of mean ^1^H ADC between low- and high-grade regions, demonstrating a statistically significant difference (*n* = 5 low-grade, *n* = 7 high-grade). (**B**) Quantitative low- and high-grade comparison between percentage areas stained positive for Ki-67 nuclear stain (*n* = 5 low-grade, *n* = 7 high-grade). ***p < 0.005.

Tissue samples harvested after imaging also demonstrated differences in proliferation and hypoxia between high- and low-grade tumors, reflected in Ki-67 proliferation marker and pimonidazole (PIM) hypoxia marker staining (Figure 2D–2E). The ^1^H ADC in high-grade tumors was significantly lower than in low-grade tumors (*p* = 0.00252, Figure 3A), likely reflecting the higher cellularity of high-grade prostate cancer, and the intensity of Ki-67 was significantly greater (*p* = 0.000666, Figure 3B). PIM staining was not statistically significant between low- and high-grade lesions due to one outlier (*p* = 0.103 including outlier, *p* = 0.0104 without; Supplementary Figure 1).

### Hyperpolarized imaging of glycolysis

**Figure 4:**
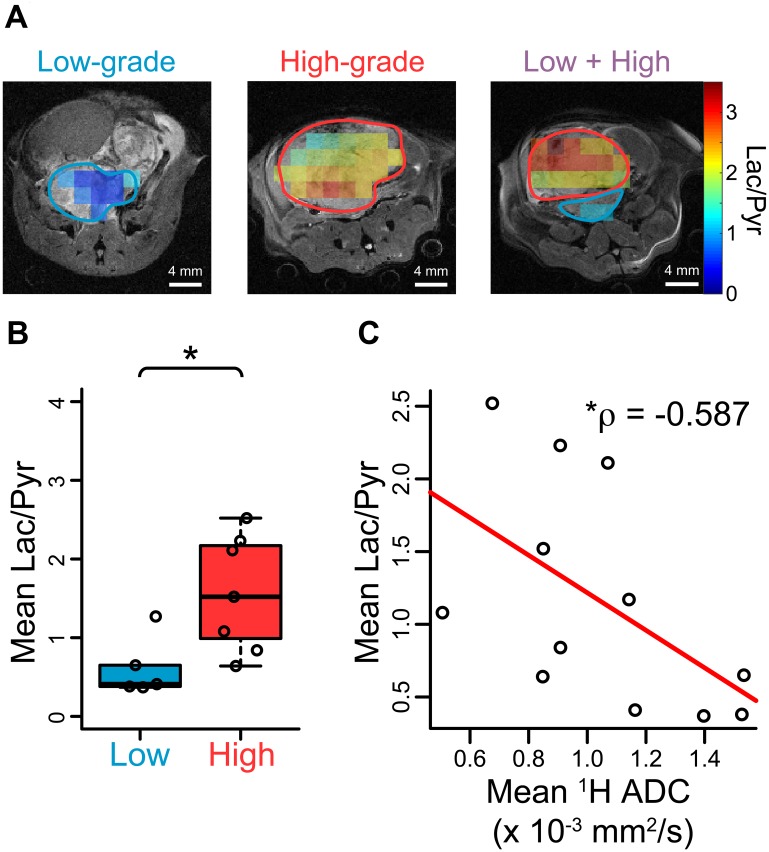
Hyperpolarized [1-^13^C] pyruvate imaging in TRAMP mice confirms glycolytic differences between low- and high-grade cancers. (**A**) Representative overlays of hyperpolarized lactate-to-pyruvate ratio (Lac/Pyr) for the mice displayed in Figure 2. (**B**) Scatter plots demonstrating a significant difference in mean Lac/Pyr ratio obtained from HP ^13^C images between low- and high-grade regions over all tumors (*n* = 5 low-grade, *n* = 7 high-grade). (**C**) The mean Lac/Pyr ratio demonstrated a negative correlation with ^1^H ADC for each tumor that was statistically significant (*p* = 0.0488). *p < 0.05.

Because pH_e_ may be dependent upon upregulation of glycolytic metabolism, we sought to quantify metabolic differences between low- and high-grade lesions. Representative spatial maps of quantified lactate-to-pyruvate (Lac/Pyr) ratio from HP ^13^C MR imaging overlaid upon conventional ^1^H anatomical images of TRAMP tumors are shown in Figure 4A. The raw magnitude images corresponding to these overlays can be found in Supplementary Figure 2A. Based upon histological staining, regions of interest on images could be classified as low- or high-grade for statistical analysis. High-grade tumor regions generally displayed higher values of Lac/Pyr compared with low-grade regions. Imaging statistics across all mouse tumor regions are compared between low- and high-grade in Figure 4B, revealing significant differences in mean Lac/Pyr ratio (*p* = 0.0303). The Lac/Pyr ratio demonstrated a moderately strong negative correlation with ^1^H ADC (Figure 4C), indicating that higher tumor cellularity may contribute to higher observed lactate conversion. Low- and high-grade tumors also demonstrated a significant difference in regional-maximum Lac/Pyr voxel ratio (p = 0.0101, Supplementary Figure 2B). We also performed quantitative RT-PCR in the same mice on tumor tissue excised after imaging in order to quantify expression of the *Ldha* gene (Supplementary Figure 2C). *Ldha* gene expression was lower in low-grade lesions, but not statistically significant (p = 0.0732). However, the two low-grade lesions that exhibited the highest *Ldha* expression came from the two mice that also included a high-grade lesion.

### Hyperpolarized imaging of extracellular acidification

**Figure 5:**
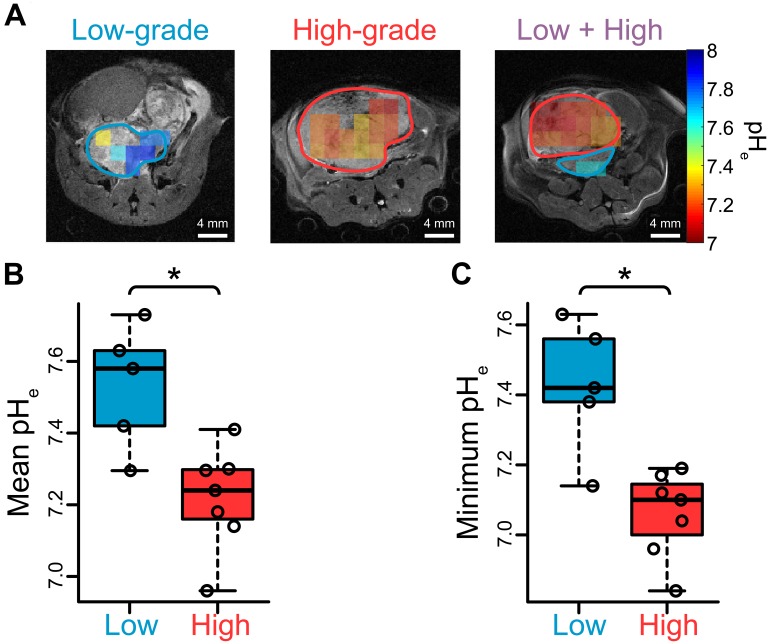
Extracellular pH measurements in TRAMP tumors via hyperpolarized [^13^C] bicarbonate imaging suggest extracellular acidification is associated with high-grade disease. (**A**) Representative overlays of extracellular pH measured with hyperpolarized [^13^C] bicarbonate for the mice displayed in Figure 2. (**B**–**C**) Scatter plots demonstrating significant differences in pH metrics obtained from HP ^13^C images between low- and high-grade regions over all mice: (B) mean; and (C) regional-minimum (*n* = 5 low-grade, *n* = 7 high-grade). *p < 0.05.

We investigated whether *in vivo* hyperpolarized pH imaging with [^13^C] bicarbonate could distinguish low- and high-grade tumor tissue. Figure 5A displays representative pH overlays obtained in the same imaging study as the hyperpolarized [1-^13^C] pyruvate imaging. The raw spectra and magnitude images corresponding to these overlays can be found in Supplementary Figure 3. Low- and high-grade tumor regions were differentiated by both their mean and regional-minimum pH values (*p* = 0.0177 and 0.0101, respectively, Figure 5B–5C). For low-grade tumors we found a mean and minimum pH of 7.53 ± 0.17 and 7.43 ± 0.19, respectively, and for high-grade tumors we found a mean and minimum pH of 7.22 ± 0.14 and 7.06 ± 0.12, respectively. Only one low-grade lesion overlapped with the high-grade distribution when looking at regional-minimum pH (Figure 5C). The tumor tissue close to the minimum pH voxel in this lesion had a histology index of 1.7 (Supplementary Figure 4), which was close to the cutoff value of 2 between low- and high-grade tumors and higher than the mean histology index of all low-grade lesions in the study (Supplementary Table 1).

### Correlation of multi-probe hyperpolarized imaging and gene expression results

**Figure 6:**
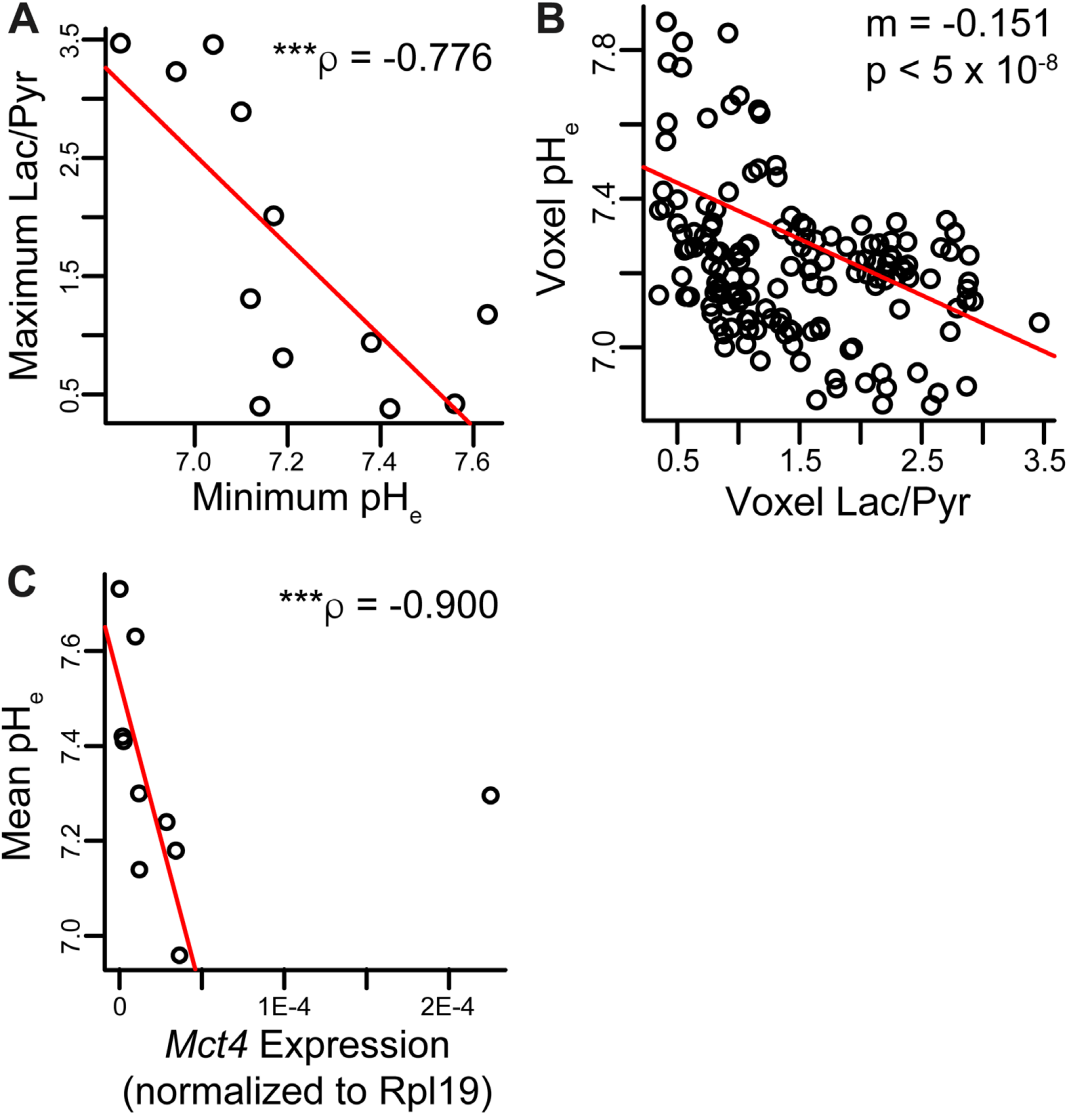
Correlations between metabolic parameters and pH_e_. (**A**) Spearman non-parametric regression between maximum Lac/Pyr and minimum pH_e_ in each ROI showed a strong negative correlation (*n* = 12 lesions). (**B**) The measured Lac/Pyr ratio and pH_e_ in each imaging voxel across all mice were negatively correlated. The pH decreased by about 0.15 unit per unit increase in Lac/Pyr ratio (*n* = 153 voxels). (**C**) Spearman non-parametric regression between mean pH and *Mct4* gene expression was strongly negative and statistically significant (*n* = 10 lesions). Two of the twelve lesions imaged had *Mct4* gene expression levels below the threshold of quantification; these lesions had average pH_e_ values of 7.30 and 7.56. ρ and *p*-value in plot exclude the outlier; the correlation was still significant with outlier included (*p* = 0.00824). ^***^ *p* < 0.005.

We investigated correlations between pH_e_ and lactate production based on HP imaging. The minimum voxel pH and maximum Lac/Pyr voxel ratio within each lesion demonstrated a strong negative correlation (ρ = –0.776, *p* = 0.00466, Figure 6A). There was a weak, non-statistically significant inverse correlation between mean imaging pH_e_ and mean Lac/Pyr ratio per lesion (ρ = –0.469, *p* = 0.128; Supplementary Figure 5A). Pooling imaging voxels across all lesions together and fitting to a linear mixed-effects model, the pH_e_ was found to be inversely correlated with the Lac/Pyr ratio in the same voxel with slope -0.151 pH unit/unit increase in Lac/Pyr ratio (*p* = 2.45 × 10^−8^; Figure 6B). The correlation between mean tumor pH_e_ and *Mct4* gene expression was strongly negative (ρ = –0.806, *p* = 0.00824; Figure 6C), suggesting that increased MCT4 expression is associated with decreased pH_e_ in TRAMP tumors. There was an inverse correlation between mean pH_e_ and *Mct1* gene expression, although statistical significance was not observed (ρ = –0.497, *p* = 0.104; Supplementary Figure 5B).

We also investigated whether there were grade-dependent differences in expression of the *Mct1*, *Mct4*, and *Hif1*α genes. All three genes were significantly overexpressed in high-grade tissue compared with low-grade (*p* = 0.00252, 0.0333, and 0.0480 for *Mct1*, *Mct4* and *Hif1*α, respectively; Supplementary Figure 6).

All imaging parameters demonstrated a large effect size between low- and high-grade lesions (|Cohen’s d| > 1.5, Supplementary Table 2). Supplementary Figure 7 shows the distributions of mean ^1^H ADC, mean HP Pyr/Lac, and mean/minimum HP pH between low- and high-grade lesions when including only one imaging region of interest (ROI) per mouse. Differences between low- and high-grade imaging metrics were still statistically significant (*p* < 0.01 for ^1^H ADC; *p* < 0.05 for all others).

**Figure 7:**
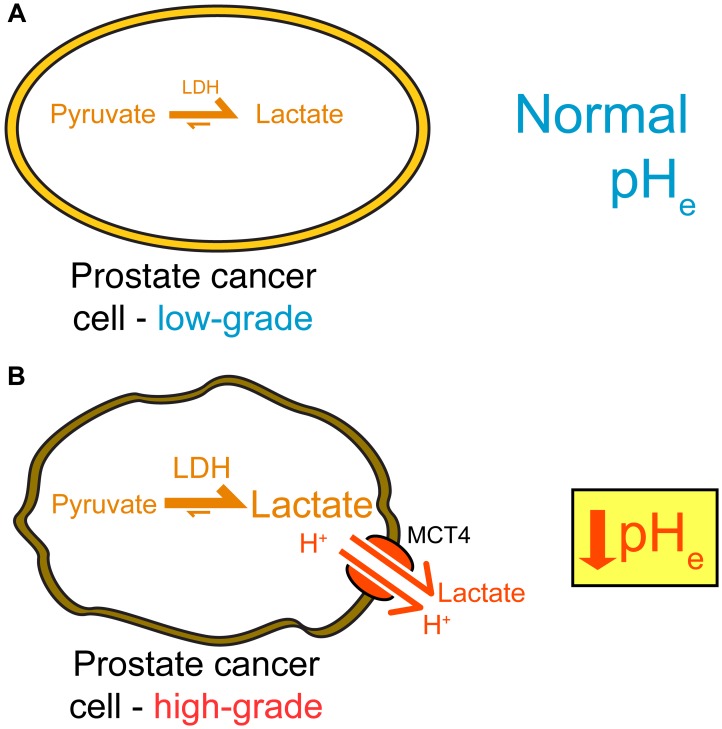
Summary of imaging and gene expression findings in TRAMP tumors. High-grade disease exhibits a metabolic shift and extracellular acidification, which can both be detected through imaging with hyperpolarized ^13^C methods. (**A**). Low-grade tumors demonstrate low levels of LDH activity, low lactate production, and a normal pH_e_. (**B**) High-grade TRAMP tumors upregulate LDHA, produce increased lactate, upregulate MCT4, and have a lower pH_e_.

## DISCUSSION

Extracellular pH is reduced in many cancers, both in humans and in animal models [17], and for many years pH electrodes have served as the predominant technology available for tissue pH measurement [17]. This is not a feasible option in the clinic due to its invasiveness as well as the possibility of perturbing the native pH environment during measurement. Changes in pH_e_ have also been demonstrated to modulate cancer cell motility and aggressive phenotype [21]. There is a growing body of literature that also suggests that spatiotemporal changes in pH heterogeneity, measurable via molecular imaging techniques, may confer a survival advantage upon cancer cells in various aspects: therapeutic resistance, immune evasion, maintaining stemness and differentiation capacity, and extracellular matrix remodeling [32]. Furthermore, pH and metabolism are known to be closely connected and may affect one another. Therefore, a clinically translatable MRI protocol that can measure pH_e_ simultaneously with metabolic markers may significantly improve clinical care of patients with prostate cancer.

In this study, we simultaneously assessed glycolysis and extracellular acidification in the TRAMP mouse in a single hyperpolarized imaging session and drew important links between the two. We have previously demonstrated that HP [^13^C] bicarbonate can be generated via rapid breakdown of HP [1-^13^C]1,2-glycerol carbonate, and we showed that we could detect pH differences between benign and tumor tissue in a TRAMP mouse [22]. A notable advantage of this technique is the elimination of toxic Cs^+^ as a counter-ion to improve bicarbonate solubility in the formulation, as has been used in previous HP reports [25, 33]. Our research group has also shown in a dual-agent HP study applying HP [1-^13^C] pyruvate and [^13^C] urea in low- and high-grade TRAMP tumors that TRAMP mice recapitulate key metabolic and histologic features of human prostate cancer. HP [^13^C] urea does not undergo metabolic conversion and therefore can be used to study tumor tissue perfusion via dynamic imaging. The study demonstrated a metabolism-perfusion mismatch in high-grade TRAMP tumors, in which regions of high HP lactate-to-pyruvate conversion demonstrated low area under the curve signal of HP urea [30]. Although the authors suggested that the high glycolytic conversion rate and low perfusion would likely contribute to extracellular acidification, they did not measure this in their cohort of TRAMP mice. The same two HP agents were utilized in a subsequent study along with multiparametric ^1^H MRI in a different cohort of TRAMP mice displaying normal prostate, low-grade tumors, and high-grade tumors [14]. Similarly, the authors observed a higher HP lactate-to-pyruvate ratio as well as a lower HP urea signal in high-grade tumors compared with both low-grade and normal prostate. A lower pH_e_ was once again implied but not explicitly measured in this cohort of TRAMP mice. In this study, we have included *in vivo* hyperpolarized [^13^C] bicarbonate pH imaging in a new cohort of low- and high-grade TRAMP tumors and confirmed that pH_e_ is significantly reduced in aggressive tumors (7.06 for high-grade versus 7.43 for low-grade).

One of the major findings of this work was that both mean and regional-minimum tumor pH obtained via imaging varied significantly between low- and high-grade lesions (Figure 5B–5C). Within the two mice that exhibited separate low- and high-grade tumor regions, the ROI mean and minimum pH values were both lower in the high-grade lesion, enabling separation of benign and aggressive cancer within the same mouse. This is similar to the human prostate, which may demonstrate a mixture of low- and high-grade cancer in various regions throughout the organ. A minimum-pH quantification scheme is encouraging in that it may be more robust to variation in ROI definition than a mean-pH quantification scheme. It may also demonstrate greater sensitivity to the presence of high-grade cancer within an imaging ROI, considering that greater acidity is closely linked with a more aggressive phenotype in cancer [21, 34]. In support of this rationale, the minimum-pH quantification scheme was able to identify a “biopsy site” within a lesion that was grossly characterized as low-grade, revealing a region of predominantly moderately-differentiated tumor cells (Supplementary Figure 4). Thus, it is likely in this case that hyperpolarized pH imaging was able to identify a lesion in this mouse that was on the cusp of developing into aggressive cancer. Our other results are in agreement with previous experimental findings. Compared with low-grade TRAMP tumors, high-grade tumors demonstrated higher proliferation and hypoxia [14, 30], a lower mean ^1^H ADC [14], altered hyperpolarized lactate imaging parameters [8, 14, 30, 35], and upregulated gene expression of glycolytic enzymes and transporters, including *Ldha*, *Mct1/4*, and *Hif1*α [14, 30]. Unlike the human situation, the mean ADC of the TRAMP tumors showed very good separation between low- and high-grade lesions (Figure 3A), which likely reflects the dramatic differences in cellularity observed histologically (Figure 2C).

We observed a strong negative correlation between mean pH and *Mct4* gene expression in the lesions studied (Figure 6B), implicating the MCT4 transport protein, which exports lactic acid, as a contributor to extracellular acidosis. There was one outlier: a lesion with very high *Mct4* expression but an alkaline mean pH_e_ value of 7.30. This lesion was smaller in volume that other high-grade lesions, and therefore the high HP signal arising from nearby vascular voxels may have spilled over, causing the lesion pH_e_ to appear more alkaline. In contrast with *Mct4*, we did not detect as strong of a correlation between pH and the *Mct1* gene (Supplementary Figure 5B), reflecting the specific association of the MCT4 transporter with high lactate efflux [36–39]. We also detected a statistically significant correlation between pH_e_ and lactate production as measured by hyperpolarized imaging, both on a per-lesion basis (Figure 6A) and on a per-voxel basis (Figure 6B). We did not observe a perfect overlap between high lactate production and low pH_e_, but this is not unexpected given the known complexity of extracellular acidity, which is a consequence of transport of protons via multiple transporters [18], interstitial buffering capacity [40], proton exchange kinetics and motility [41], and tissue perfusion [42]. We present a model of TRAMP tumor progression in Figure 7 that summarizes the observed lactate, pH, and gene expression correlations. In low-grade tumors, there is relatively little production of lactate as measured with hyperpolarized [1-^13^C] pyruvate imaging [8, 14, 30] (Figure 4) and a normal pH_e_ of 7.43 (Figure 5). In contrast, high-grade tumors demonstrate increased production of lactate (Figure 4) by lactate dehydrogenase [8, 14, 30], increased expression of MCT4 [14, 30], and a decreased pH_e_ of 7.06 (Figure 5). This model reflects the correlations observed between increased lactic acid production, decreased extracellular pH, and disease grade in the TRAMP model.

Several limitations of our study design are worth mentioning. First, the coarse spatial resolution of hyperpolarized pH imaging (4 mm in-plane, 6.5 mm slice) meant that some voxels contained a mixture of various tissue types, including benign and aggressive tumor as well as stromal tissue. Nevertheless, this resolution was sufficient for us to capture tumor pH heterogeneity. Advances in hyperpolarization and MR imaging may be able to reduce the achievable voxel size and thus better isolate tissue components. Second, our study cohort of mice was too small (n = 10) to detect significant differences in PIM staining and *Ldha* gene expression as in prior literature [14, 30]. However, the two low-grade tumors with the highest *Ldha* expression came from the two mice with high-grade lesions as well, perhaps due to the unintentional inclusion of nearby high-grade tissue in the sample. Despite the small cohort size, differences in imaging parameters between low- and high-grade tumors, and the majority of the gene expression data, demonstrated statistical significance. Third, we did not include mice with normal prostates in our study, primarily because we did not have sufficient imaging spatial resolution to sufficiently capture it. This does not change the significance of the finding that low- and high-grade tumors demonstrate significantly different pH_e_ values. Finally, all tumor regions were treated as independent samples, even though some regions analyzed came from the same mouse. In order to correct for this potential source of error, we performed a per-mouse analysis and still detected significant differences in mean ^1^H ADC, mean HP Pyr/Lac, and mean/minimum HP pH (Supplementary Figure 7).

Hyperpolarized pH imaging may significantly improve clinical management of prostate cancer. Current standard-of-care prostate cancer multiparametric MRI includes T_2_- and diffusion-weighted imaging and dynamic contrast-enhanced imaging, interpreted using the Prostate Imaging Reporting & Data System (PI-RADS) framework [43–45]. This method is highly effective in detecting prostate cancer and localization for subsequent biopsy, but it can miss small volumes of high-grade disease and fail to reliably grade lesions based on imaging characteristics [4, 46–48]. In particular, although ^1^H diffusion-weighted imaging is sensitive to detect highly cellular late-stage lesions [49], false positives can arise from tissue compression or high stromal composition [50]. Furthermore, imaging parameters can be compromised by biopsy of the imaging site [50]. We are unaware of other pathological conditions that may cause interstitial acidification in prostate cancer and confound HP pH_e_ imaging results. In contrast, a high degree of alkalinization of human prostatic fluid has been observed with prostatic infection [51]. Hyperpolarized [1-^13^C] pyruvate imaging has been applied in men with prostate cancer, with the key finding of increased lactate production in tumors [10]. It is worth noting that HP imaging not only can be completed more quickly than other types of metabolic MRI (one or two minutes compared with 10–30 minutes), but it also boasts the potential capability of imaging multiple HP agents simultaneously in a single scan [11]. We have extended hyperpolarized *in vivo* imaging beyond pyruvate alone to also include imaging of [^13^C] bicarbonate in the same imaging session, aided by recent advances in the underlying polarization techniques and chemistry [22].

The HP bicarbonate imaging method utilized in this study holds promise for eventual clinical translation. While other HP compounds have demonstrated promise for measuring pH_e_ *in vivo*, including N-(2-acetamido)-2-aminoethanesulfonic acid (ACES) [26], diethylmalonic acid (DEMA) [27], zymonic acid [52], and amino acid derivatives [52], bicarbonate is routinely infused into patients at high concentration and therefore has a low barrier to entry. Nevertheless, significant challenges exist. One prominent challenge is the short *in vivo* spin-lattice relaxation time constant (T_1_) of [^13^C] bicarbonate (10 s) [11, 25]. Another is the relatively slow kinetic conversion between bicarbonate and CO_2_, which requires sufficient time to reach equilibrium pH and depends upon expression and localization of carbonic anhydrase (CA) isoforms. We have previously measured the characteristic exchange rate constant for bicarbonate-CO_2_ to be 1.56 s^-1^ in high-grade TRAMP tumors [53], which suggests that pH equilibrium is reached (95%) in ~2 s. Bicarbonate-CO_2_ exchange might be slower in low-grade tumors if extracellular CA isoforms are not as strongly expressed, although we have not measured either exchange or CA expression in low-grade tumors. Some HP bicarbonate and CO_2_ in a given voxel may also distribute between erythrocytes, blood plasma, and tumor cytoplasm, meaning that the measured pH is not strictly extracellular. These effects, individually or in combination, may have given rise to the unusually high pH_e_ values observed in low-grade lesions (Figure 5B). Despite these challenges, the GLC method of [^13^C] bicarbonate production used in this manuscript enables production of a polarized solution, similar to pyruvate, when prepared with the same methods (18% for pyruvate vs. 19% for bicarbonate). Moreover, the method produced high quality, high-resolution maps of pH_e_ (Figure 5). Finally, recent advances in pulse sequence acquisition promise to increase the signal-to-noise ratio of this method [53]. Taken together, these observations suggest the potential feasibility and utility of using hyperpolarized ^13^C pH_e_ imaging for detecting aggressive disease in men with localized prostate cancer.

In conclusion, we conducted hyperpolarized imaging in transgenic prostate cancer mice in order to simultaneously obtain measures of glycolytic metabolism and pH_e_. We observed significant differences in both Lac/Pyr ratio and pH_e_ between histologically classified low- and high-grade tumors, consistent with increased expression of glycolytic enzymes and transporters in high-grade compared with low-grade tissue samples. These results suggest that hyperpolarized pH_e_ imaging may be able to stratify low-risk and high-risk prostate cancer patients in a non-invasive exam.

## MATERIALS AND METHODS

Additional experimental details may be found in the Supplementary Materials.

### Chemicals and ^13^C imaging agent formulations

[1-^13^C]1,2-glycerol carbonate (GLC) was purchased from Cambridge Isotopes Laboratories, Inc. (Tewksbury, MA, USA). [1-^13^C] pyruvic acid was purchased from Isotec (Miamisburg, OH, USA). The OX063 and GE trityl radicals were purchased from Oxford Instruments (Abingdon, UK). Other chemicals and solvents were purchased from Aldrich (St. Louis, MO, USA). GLC was formulated for hyperpolarization as previously described [22] and consisted of 15 mM OX063 radical dissolved in the neat liquid. [1-^13^C] pyruvic acid was also formulated as previously described [11].

### Animal protocol and handling

All animal studies were conducted in accordance with the policies of the Institutional Animal Care and Use Committee (IACUC) at the University of California, San Francisco. Transgenic adenocarcinoma of the mouse prostate (TRAMP) model mice [29] were supplied by Roswell Park Cancer Institute (Buffalo, NY, USA). This mouse model was chosen because it is known to recapitulate features of human prostate cancer that are relevant to this study, including progression from a low-grade to a high-grade phenotype, as characterized by histopathology [31], as well as a metabolic switch to glycolytic metabolism in higher-grade disease [8, 14, 30, 54]. Mice were cannulated in the lateral tail vein and anesthetized with 1-2% isoflurane/100% oxygen at a rate of 1 L/min. The anesthetized mouse was secured in an MR-compatible holder with a 37° C water pad and placed within the vertical bore of the imaging system. Every 10–12 minutes, the catheter was flushed with 8 μg/mL heparin in normal saline to prevent clotting. The mouse was given at least 15 minutes of recovery time between subsequent injections.

### Animal MR imaging protocol

All MR imaging was performed on a vertical-bore 14 T Varian MR imaging system (150 MHz ^13^C, Varian Instruments). ^1^H diffusion-weighted spin-echo axial images covering the entire tumor volume (40 × 40 mm^2^ [128 × 128 matrix] in-plane, 1 mm slice thickness, 0.25 mm slice spacing, 4 b-values between 0 and 515 s/mm^2^) were acquired using a 40 mm ^1^H quadrature millipede coil (Agilent Technologies, Palo Alto, CA, USA). All other imaging data were acquired with a 40 mm dual-tuned ^1^H/^13^C quadrature birdcage coil (m2m Imaging, Cleveland, OH). ^1^H axial and coronal images were acquired either prior to hyperpolarized injections or inbetween. Each mouse received two injections of HP ^13^C imaging agent: 100 mM [^13^C] bicarbonate produced via rapid hydrolysis of HP [1-^13^C] GLC, as previously described [22], and 80 mM sodium [1-^13^C] pyruvate in 40 mM TRIS buffer. Both agents were polarized using a HyperSense preclinical polarizer (Oxford Instruments, Abingdon, UK). The mean back-calculated polarization values for [1-^13^C] pyruvic acid and [^13^C] bicarbonate (generated from [1-^13^C] GLC) were 18% and 19%, respectively. Approximately 450 μL of agent were injected via the tail vein catheter over 15 s for pyruvic acid and 12 s for [^13^C] bicarbonate. For the [^13^C] bicarbonate injection, a 2D chemical shift imaging (2D CSI) sequence was performed 16–18 s after the start of injection (8 × 8 × 256 matrix size, 6.5 or 10 mm axial slice thickness, centric phase encoding, FOV = 32 × 32 mm^2^, 8013 Hz spectral width, TR = 67 ms, total imaging time ~4 s). To avoid chemical shift misregistration between bicarbonate/CO_2_ slices, a two-band Gaussian excitation pulse was used, which delivered nominal tip angles of 25° and 2.78° to CO_2_ and bicarbonate, respectively [22]. The ratio of signal excitation between the two pulse bands, necessary for calculating pH maps, was empirically determined beforehand using a spherical [^13^C] urea phantom. For the [1-^13^C] pyruvate injection, a single-shot 3D gradient-spin echo (3D GRASE) sequence [55] was used to obtain metabolite maps of pyruvate and lactate (6 ms SLR excitation + refocusing pulses, 40 × 40 × 40 mm^3^ FOV, 12 × 12 × 16 matrix, center-out encoding in 2nd phase-encode dimension, total time/metabolite = 156 ms). The 3D GRASE sequence was initiated 36 s after the start of injection.

### Image processing

More details are available in the Supplementary Materials. The ^1^H apparent diffusion coefficient (ADC) maps were generated along with root mean square (RMS) residuals using VnmrJ 4.2A software (Agilent Technologies, Palo Alto, CA, USA). The 2D CSI ^13^C imaging data were processed using SIVIC open-source software (Sourceforge. net) [56]. All other image-processing steps, described below, were performed using custom MATLAB scripts (MathWorks, Natick, MA, USA).

Calculated ADC maps from ^1^H diffusion-weighted imaging were thresholded using an upper limit of 500 for the RMS residual. All volume elements (voxels) with ADC > 3.0 × 10^-3^ mm^2^/s, the diffusion coefficient of free water at 37 °C [57], were also excluded from analysis. ROIs were then drawn on each ^1^H imaging slice, with each ROI being designated as either low-grade or high-grade tumor, based on histology. Low-grade and high-grade ROI data were combined across both mice, and the ADC values were extracted from the ROIs and used for statistical analysis.

HP image reconstruction was conducted similar to prior reports [14]. Briefly, all HP images were reconstructed to an in-plane spatial resolution of 2 × 2 mm^2^ and aligned. Axial slices of the pyruvate and lactate 3D GRASE images were averaged together in order to match the 2D CSI slice thickness for bicarbonate/CO_2_. The lactate magnitude images were thresholded to exclude voxels with SNR < 4, whereas pyruvate voxels with SNR < 4 were set to the mean noise magnitude. Lactate-to-pyruvate ratio (Lac/Pyr) maps were then calculated from the pyruvate and lactate magnitude images. [^13^C] bicarbonate and CO_2_ magnitude peak integral images were thresholded to exclude voxels with SNR < 3, and the intensities were corrected based upon the difference in tip angle before pH calculation using a modified Henderson-Hasselbalch equation [22].

Voxels for analysis were selected using the axial and coronal anatomical images and designated as part of either a low-grade or high-grade ROI. Only voxels comprised of at least 50% tumor based on ^1^H anatomical images and histology were included.

### Histological staining and qRT-PCR

Mice were euthanized and dissected within 48 hours of imaging. For hypoxia staining, mice were injected with 60 mg/kg pimonidazole-HCl 45-60 minutes prior to euthanasia. During dissection, visually distinct tumor tissue samples were identified by a uro-oncologist (R. B.) highly experienced in murine pathology based upon tissue coloration, texture, density, and morphological distortion distinguishing them from normal genitourinary organ structure. Tissue samples were excised with their relative location noted (anterior/posterior, lateral side, ventral/dorsal with reference to normal genitourinary organs including urethra and seminal vesicles) and/or photographed for matching later on with anatomical MR images. Excised tumor tissue samples were immediately fixed in 10% buffered formalin or flash-frozen with liquid nitrogen. Tissue staining was performed on 4 μm-thick sections using hematoxylin and eosin (H&E, Thermo Scientific, Waltham, MA, USA), anti-Ki-67 (Cell Signaling Technology, Danvers, MA, USA), or anti-pimonidazole mouse IgG1 monoclonal antibody (Hypoxyprobe Inc., Burlington, MA, USA) on step sections. For very large tumors extending beyond the axial slice thickness of the ^13^C imaging, the tissue section used for histology did not necessarily correspond with the MR imaging volume. A histologic index for each tissue sample was calculated, similar to previous studies [8, 14, 30], based on the weighted percentages of tumor differentiation (normal, well-differentiated, moderately well-differentiated and poorly-differentiated) from standard H&E staining. The histologic index ranged between 0 and 3, where 0 indicated that 100% of the tissue was normal and 3 indicated that 100% of the tissue was poorly differentiated. Each tumor received a gross histological index by calculating the mean of all matched tissue samples, then was dichotomized to be either low-grade (index ≤ 2) or high-grade (index > 2), as previously described [14], in a manner that mimics the clinical pathologic practice in which patients with Gleason score ≤ 3+3 are considered to have low-grade compared to Gleason ≥ 3+4 high grade tumors.

The degree of Ki-67 and pimonidazole staining was determined for each tumor as follows. The approximate percentage of cells stained positive for Ki-67 in viable tumor areas across an entire, representative cross-sectional specimen of each tumor was assessed visually by an experienced TRAMP pathologist, taking multiple, high-power/small-field cell counts from across the specimen. For pimonidazole, the percentage of positively stained cellular areas was similarly obtained across an entire, representative cross-section specimen of each tumor. If multiple specimens from the same tumor region were available, these Ki-67 and pimonidazole percentages were averaged.

For qRT-PCR, total RNA was extracted from snap-frozen tumor tissue using an RNeasy kit (Qiagen, Limburg, Netherlands) and measured using an RNA6000 Kit (Agilent, Santa Clara, CA, USA). The RNA extract was then subjected to RQ1 RNAse-free DNAse (Promega Manufacturing, Madison, WI, USA) in order to digest in-sample gDNA. Reverse transcription was performed with a qScript DNA Synthesis Kit (Quanta Biosciences, Gaithersburg, MD, USA), and subsequent quantitative RT-PCR was performed in triplicate using 96-well plates in a PikoReal real-time PCR system (Thermo Scientific, Waltham, MA, USA). qPCR probes (TaqMan, Life Technologies, Carlsbad, CA, USA) were selected for the following genes: lactate dehydrogenase subunit *Ldha*, monocarboxylate transporters (*Mct1*, *Mct4*), and hypoxia-inducible factor *Hif1*α. Expression was normalized to the 60S ribosomal protein L19 (*Rpl19*) gene in each sample.

### Statistical analysis

All statistical analyses were performed using R software (The R Foundation; for package citations please see Supplementary Materials) [58]. Mean or minimum ROI voxel values were reported for each tumor. Low- and high-grade tumors were compared using nonparametric Mann-Whitney *U* tests. Spearman correlations between data sets were estimated across all tumor samples. The correlation between the Lac/Pyr ratio and pH_e_ across all voxels was assessed by constructing a linear mixed-effects model accounting for individual lesions and mice. The Cohen’s d-statistic for effect size was calculated for all imaging parameters. Statistics were reported in the text as mean ± standard deviation. A *p*-value less than 0.05 was considered statistically significant.

## SUPPLEMENTARY MATERIALS



## Abbreviations

pH_e_: Extracellular pH
HP: Hyperpolarized
TRAMP: Transgenic adenocarcinoma of the mouse prostate
ADC: Apparent diffusion coefficient
MR: Magnetic resonance
MRI: Magnetic resonance imaging
DNP: Dynamic nuclear polarization
PIM: Pimonidazole
LDH: Lactate dehydrogenase
LDHA: Lactate dehydrogenase, subunit A
MCT1: Monocarboxylate transporter 1
MCT4: Monocarboxylate transporter 4
HIF1α: Hypoxia-inducible factor 1a
RPL19: 60S ribosomal protein L19
Lac/Pyr: Lactate-to-pyruvate ratio
ROI: Region of interest
SNR: Signal-to-noise ratio
PI-RADS: Prostate Imaging Reporting & Data System
T_1_: Spin-lattice relaxation time constant
GLC: 1,2-glycerol carbonate
ACES: N-(2-acetamido)-2-aminoethanesulfonic acid
DEMA: Diethylmalonic acid
CA: Carbonic anhydrase
RMS: Root mean square
IACUC: Institutional Animal Care and Use Committee
3D GRASE: 3D gradient-spin echo
2D CSI: 2D chemical shift imaging
FOV: Field of view
TR: Repetition time
SLR: Shinnar-Le Roux
H&E: Hematoxylin and eosin
