# Supplementary material for: Hyperpolarized in vivo pH imaging reveals grade-dependent acidification in prostate cancer
Source: Oncotarget. 2019 Oct 22;10(58):6096–110. doi: 10.18632/oncotarget.27225 (PMC6817439; doi:10.18632/oncotarget.27225)
Supplement: Supplementary file 1 [file oncotarget-10-6096-s001.pdf]

Hyperpolarized *in vivo* pH imaging reveals grade-dependent acidification in prostate cancer

SUPPLEMENTARY MATERIALS

Animal MR imaging protocol

All MR imaging was performed on a vertical-bore 14 T Varian MR imaging system (150 MHz ^{13}C, Varian Instruments) equipped with either a 40 mm ^{1}H quadrature millipede coil (Agilent Technologies, Palo Alto, CA, USA) or a 40 mm dual-tuned $^{1}H/^{13}C$ quadrature birdcage coil (m2m Imaging, Cleveland, OH, USA). First, the ^{1}H-only coil was tuned and used to acquire axial and coronal images (40 × 40 mm^2 in-plane, 1 mm slice thickness, 0.25 mm slice spacing, TE/TR = 20/1200 ms) as well as ^{1}H diffusion-weighted spin-echo axial images covering the entire tumor volume (40 × 40 mm^2 [128 × 128 matrix] in-plane, 1 mm slice thickness, 0.25 mm slice spacing, 4 *b*-values between 0 and 515 s/mm^2). Localized gradient shimming was performed on the tumor prior to imaging. The ^{1}H-only coil was then switched out for the $^{1}H/^{13}C$ coil while keeping the same mouse positioning within the magnet, and tuning and gradient shimming were repeated. ^{1}H axial and coronal images were acquired either prior to hyperpolarized injections or between them. Prior to all ^{13}C imaging, the ^{13}C transmitter offset was calculated from the ^{1}H water resonance offset in a PRESS localized spectroscopy voxel centered on the tumor.

Each mouse received two injections of HP ^{13}C imaging agent: 140 mM [^{13}C]bicarbonate produced via rapid hydrolysis of HP [1-^{13}C]GLC, as previously described [1], or 80 mM sodium [1-^{13}C]pyruvate in 40 mM TRIS buffer. Briefly, 70 mg of [1-^{13}C]GLC were polarized, dissolved with 3.5 mL of 0.3 mM EDTA in water, mixed with 2 equivalents of NaOH (96 mg of a 50-52% NaOH solution) and heated for 10 seconds following dissolution, and finally neutralized with 575 mg of 1.5 M HCl. Both agents were polarized using a HyperSense preclinical polarizer (Oxford Instruments, Abingdon, UK). The total polarization time was 1 hour for [1-^{13}C] pyruvic acid and 3 hours for [1-^{13}C]GLC. The mean back-calculated polarization values for [1-^{13}C]pyruvic acid and [^{13}C]bicarbonate (generated from [1-^{13}C]GLC) were 18% and 19%, respectively.

Image processing

The ^{1}H apparent diffusion coefficient (ADC) maps were generated along with root mean square (RMS) residuals using VnmrJ 4.2A software (Agilent Technologies, Palo Alto, CA, USA). The k-space data were zero-filled to 256 × 256 in-plane prior to Fourier transform. The 2D CSI ^{13}C spectral imaging data were processed using SIVIC open-source software (Sourceforge.net) [2] in order to generate intensity maps. All other image-processing steps were performed using custom MATLAB scripts (MathWorks, Natick, MA, USA). The processing steps are detailed below:

^{1}H diffusion images

ADC maps were thresholded using an upper limit of 500 for the RMS residual. All voxels with ADC > 3.0 × 10^{-3} mm^2/s, which is the diffusion coefficient of free water at 37° C [3], were also excluded from analysis. ROIs were then drawn on each ^{1}H imaging slice, with each ROI being designated as either low-grade or high-grade tumor, based on histology. Low-grade and high-grade ROI data were combined across both mice, and the ADC values were extracted from the ROIs and used for statistical analysis.

HP [1-^{13}C]pyruvate images

Image analysis of maps from [1-^{13}C]pyruvate was conducted similar to prior reports [4]. Briefly, raw 3D GRASE data were zero-filled to 20 × 20 × 16, then shifted ~1/4 voxel up and left based upon ^{13}C-phantom imaging. Axial slices of the pyruvate and lactate 3D GRASE images were averaged together in order to match the 2D CSI slice thickness for bicarbonate/CO_2. The phased noise standard deviation was calculated from an ROI far from any ^{13}C signal and used for signal-to-noise ratio (SNR) calculation. The lactate magnitude images were thresholded to exclude voxels with SNR < 4, whereas pyruvate voxels with SNR < 4 were set to the mean noise magnitude. Lactate-to-pyruvate ratio (Lac/Pyr)

maps were then calculated from the pyruvate and lactate magnitude images.

HP [^{13}C]bicarbonate images

Raw data were 10 Hz apodized in the spectral domain and zero-filled to 16 × 16 spatially prior to Fourier transformation. Integration of the bicarbonate and CO_2 peaks was performed on the magnitude spectra, and overlays were generated. Both [^{13}C]bicarbonate and CO_2 images were thresholded to exclude voxels with SNR < 3, and the intensities were corrected based upon the difference in tip angle before pH calculation using a modified Henderson-Hasselbalch equation [1]. Voxels for analysis were selected using the axial and coronal anatomical images and designated as part of either a low-grade or high-grade ROI. Only voxels comprised of at least 50% tumor, as judged by analysis of ^{1}H anatomical images and histology, were selected.

Histological staining, qRT-PCR

While each mouse was dissected under anesthesia, the tumor, proximal lymph nodes, spleen, kidneys, liver, lungs, thymus, and any metastatic lesions were removed and immediately fixed in 10% buffered formalin or flash-frozen with liquid nitrogen. Tumor tissue for staining was transitioned into 10% neutral-buffered formalin and embedded in paraffin using a Leica TP1020 benchtop tissue processor (Leica Biosystems, Buffalo Grove, IL, USA), sectioned into 4 µm-thick sections using a rotary microtome (Leica Biosystems, Buffalo Grove, IL, USA), and dried on glass slides.

The TaqMan qPCR probes (Life Technologies, Carlsbad, CA, USA) for the genes investigated in this study were as follows: lactate dehydrogenase subunit *Ldha* (assay ID Mm01612132_m1), monocarboxylate transporter *Mct1* (assay ID Mm01306379_m1), monocarboxylate transporter *Mct3/4* (assay ID Mm00446102_m1), hypoxia-inducible factor *Hif1α* (assay ID Mm00468869_ m1), and *Rpl19* control gene (assay ID Mm02601633_g1).

Statistical analysis

All statistical analyses were performed using R software (The R Foundation) [5] and the following individual packages: "lme4" [6], "lmerTest" [7], and "effsize" [8]. Mean or minimum ROI voxel values were reported for each tumor. Low- and high-grade tumors were compared using nonparametric Mann–Whitney *U* tests. Spearman correlations between data sets were estimated across all tumor samples. The correlation between the Lac/Pyr ratio and pH_e across all voxels was assessed by constructing a linear mixed-effects model accounting for individual lesions and mice. The Cohen's d-statistic for effect size was calculated for all imaging parameters. Statistics were reported in the text as mean ± standard deviation. A p-value less than 0.05 was considered statistically significant.

REFERENCES

1. Korenchan DE, Flavell RR, Baligand C, Sriram R, Neumann K, Sukumar S, VanBrocklin H, Vigneron DB, Wilson DM, Kurhanewicz J. Dynamic nuclear polarization of biocompatible ^{13}C-enriched carbonates for *in vivo* pH imaging. Chem Commun. Royal Society of Chemistry; 2016; 52: 3030–3. https://doi.org/10.1039/C5CC09724J.
2. Crane JC, Olson MP, Nelson SJ. SIVIC: Open-Source, Standards-Based Software for DICOM MR Spectroscopy Workflows. International Journal of Biomedical Imaging. 2013; 2013:1–12. https://doi.org/10.1002/mrm.20663.
3. Holz M, Heil SR, Sacco A. Temperature-dependent self-diffusion coefficients of water and six selected molecular liquids for calibration in accurate ^{1}H NMR PFG measurements. Phys Chem Chem Phys. 2000; 2:4740–2. https://doi.org/10.1039/b005319h.
4. Bok R, Lee J, Sriram R, Keshari K, Sukumar S, Daneshmandi S, Korenchan D, Flavell R, Vigneron D, Kurhanewicz J, Seth P. The Role of Lactate Metabolism in Prostate Cancer Progression and Metastases Revealed by Dual-Agent Hyperpolarized ^{13}C MRSI. Cancers. Multidisciplinary Digital Publishing Institute; 2019; 11:257–18. https://doi.org/10.3390/cancers11020257.
5. R Core Team. R: A language and environment for statistical computing [Internet]. Vienna, Austria; 2018. Available from https://www.R-project.org/.
6. Bates D, Mächler M, Ben Bolker, Walker S. Fitting Linear Mixed-Effects Models using lme4. arXiv.org. 2014.
7. Kuznetsova A, Brockhoff PB, Christensen RHB. lmerTest Package: Tests in Linear Mixed Effects Models. J STAT SOFTW. The Foundation for Open Access Statistics; 2017; 82. https://doi.org/10.18637/jss.v082.i13.
8. Torchiano M. effsize: Efficient effect size computation [Internet]. 7 ed. 2018. https://doi.org/10.5281/zenodo.1480624.

Supplementary Table 1: List of tumor regions and associated histological indices

Mouse	% Normal	% Well-Differentiated	% Moderately-Differentiated	% Poorly-Differentiated	Histology Score	Tumor Grade
1	28%	71%	1%	0%	0.73	Low
2	0%	59%	39%	2%	1.43	Low
3	8%	47%	52%	0%	1.45	Low
4	12%	54%	33%	1%	1.22	Low
	0%	0%	0%	100%	3.00	High
5	0%	50%	50%	0%	1.50	Low
	0%	5%	5%	90%	2.85	High
6	0%	13%	17%	70%	2.57	High
7	0%	1%	3%	96%	2.94	High
8	0%	2%	0%	98%	2.96	High
9	0%	1%	0%	99%	2.98	High
10	0%	0%	0%	100%	3.00	High

Mean percentages are reported over all available H&E-stained sections as well as calculated histology score (= 1 * [% well-differentiated/normal] + 2 * [% moderately-differentiated] + 3 * [% poorly-differentiated]) and assigned tumor grade. Note that Mice 4 and 5 each had two lesions, one low-grade and one high-grade.

Supplementary Table 2: Calculated Cohen's d-statistic magnitude and 95% confidence interval for each imaging parameter compared between low- and high-grade lesions in this study

Imaging parameter	|Cohen's d|	95% Confidence Interval
Mean ^{1}H ADC	2.849	[1.010, 4.688]
Mean Lac/Pyr	1.582	[0.092, 3.072]
Max Lac/Pyr	2.055	[0.450, 3.660]
Mean pH	2.004	[0.412, 3.595]
Min pH	2.380	[0.685, 4.075]

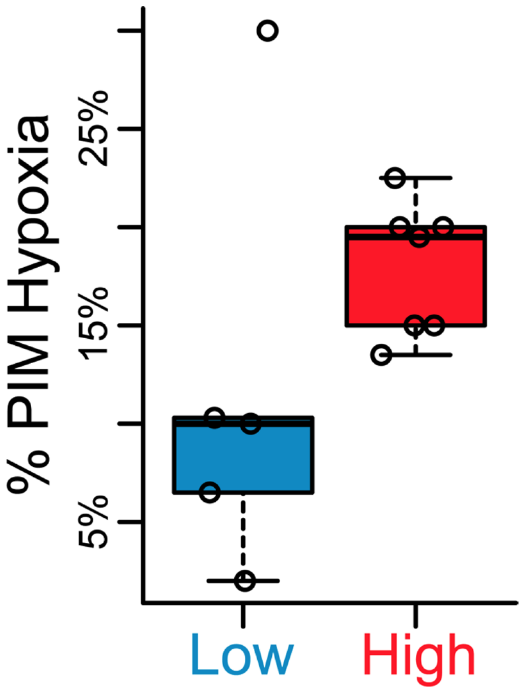

Supplementary Figure 1: Anti-pimonidazole (PIM) staining between low- and high-grade lesions. The difference approached statistical significance, $p = 0.103$ ($n = 5$ low-grade, $n = 7$ high-grade). Without the low-grade outlier, $p = 0.0104$.

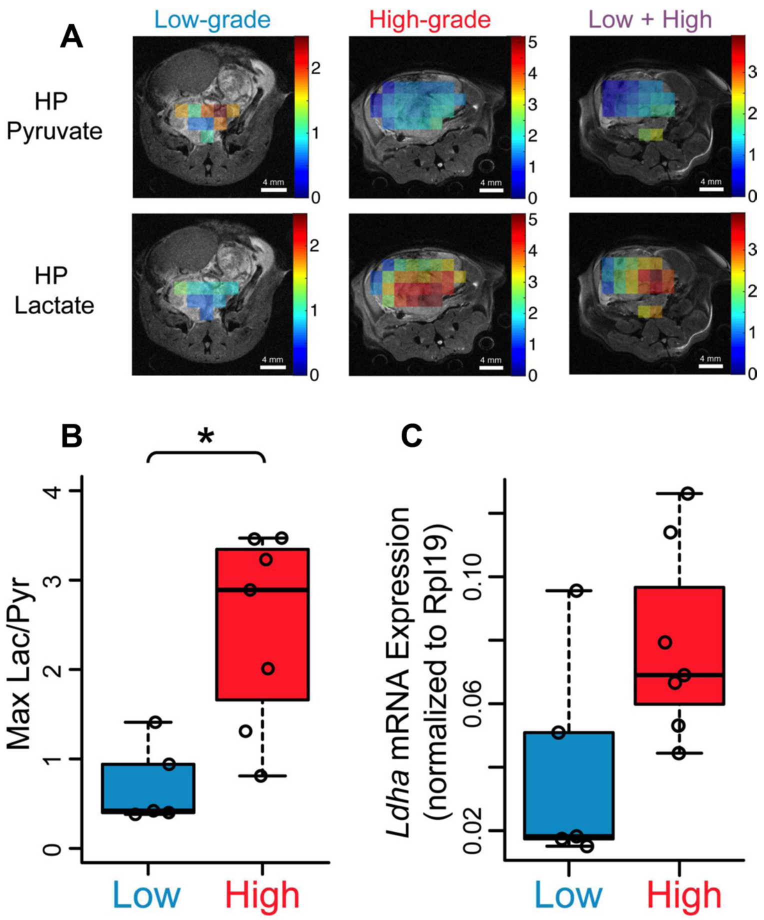

Supplementary Figure 2: Raw hyperpolarized imaging data and other measures of glycolysis. (**A**) Representative magnitude images for hyperpolarized pyruvate and lactate overlaid on ^{1}H anatomical images for the same mice shown in Figure 4 in the main manuscript. Overlay intensity is in arbitrary units. (**B**) Analysis of low- and high-grade tumor ROIs based upon the maximum voxel Lac/Pyr ratio ($n = 5$ low-grade, $n = 7$ high-grade). (**C**) *Ldha* gene expression between low- and high-grade tissue samples across all tumors approached statistical significance, $p = 0.0732$ ($n = 5$ low-grade, $n = 7$ high-grade).

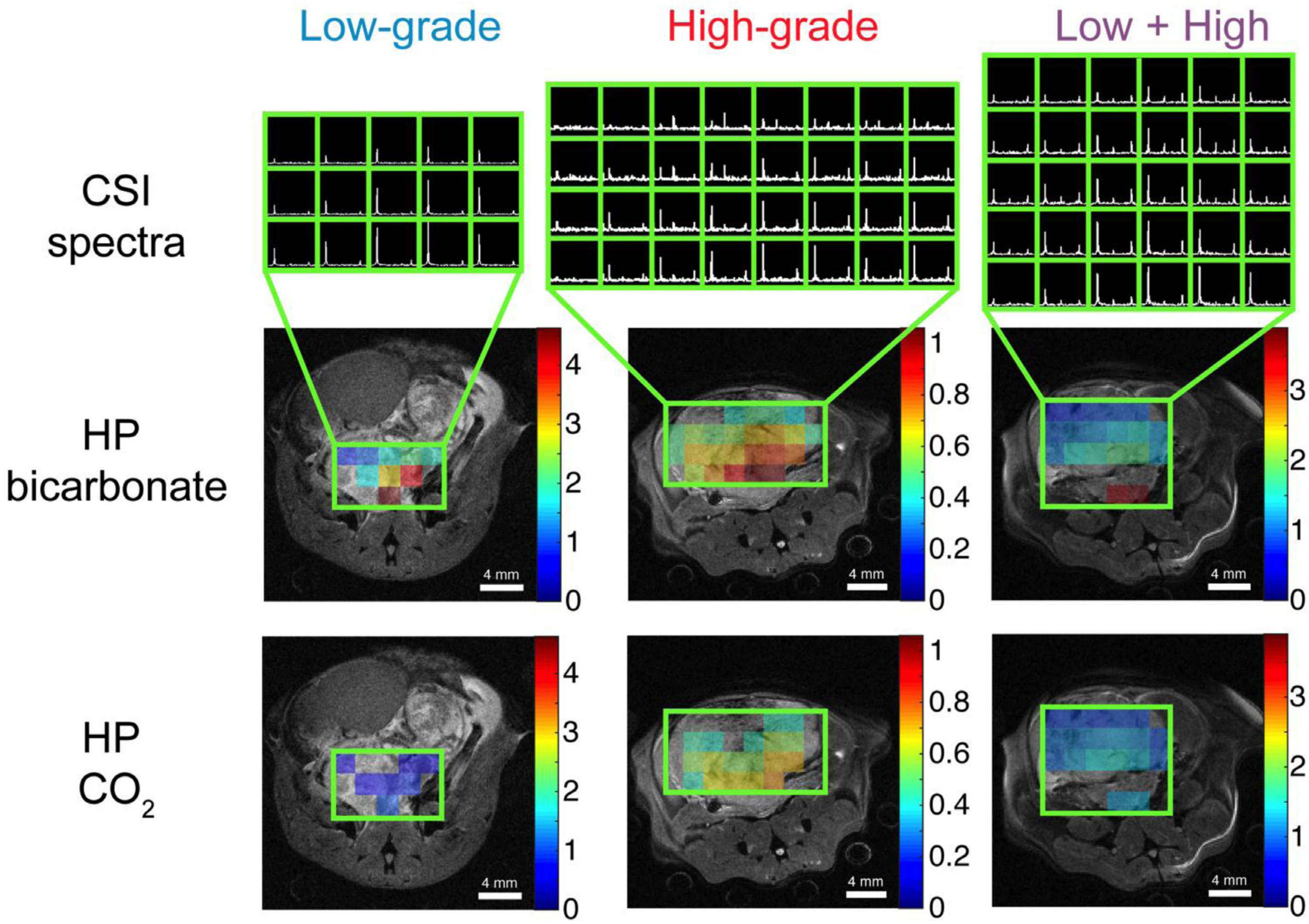

Supplementary Figure 3: Raw imaging data for hyperpolarized pH imaging. Representative 2D CSI spectra obtained from hyperpolarized [^{13}C]bicarbonate imaging, as well as magnitude integral images of the bicarbonate and CO_2 peaks. In each spectrum, the downfield (leftmost) peak is bicarbonate, and the upfield (rightmost) peak is CO_2. Some spectra demonstrate a transmitter glitch in the center of the spectrum. Mice shown here are the same as in Figure 5 in the main manuscript. Green boxes on ^{1}H overlays show the region corresponding to the spectra. Overlay intensity is in arbitrary units.

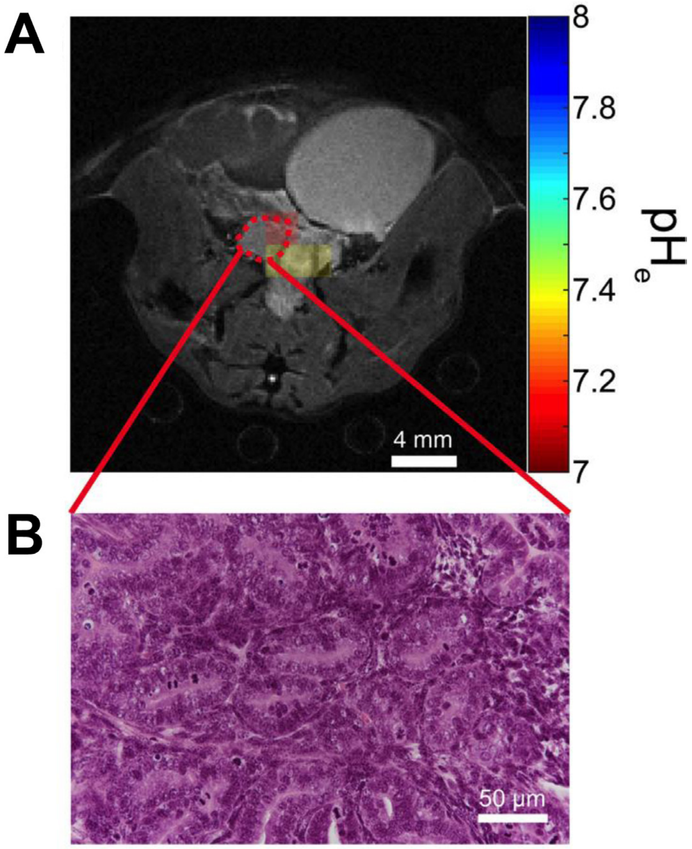

Supplementary Figure 4: Analysis of the low-grade lesion with the lowest minimum voxel pH. This figure pertains to mouse 3 in Supplementary Table 1. (**A**) pH_e voxel values measured using hyperpolarized [^{13}C]bicarbonate imaging overlaid on a T_2-weighted ^1H anatomical image. Red dotted outline indicates tissue region that was resected for histological staining. (**B**) Representative image depicting H&E tissue stain of tumor nodule indicated in (A). The tissue was characterized as being comprised of 30% normal or well-differentiated cells and 70% moderately-differentiated cells, corresponding with a histological index of 1.7 that was close to the cutoff of 2 between low- and high-grade lesions.

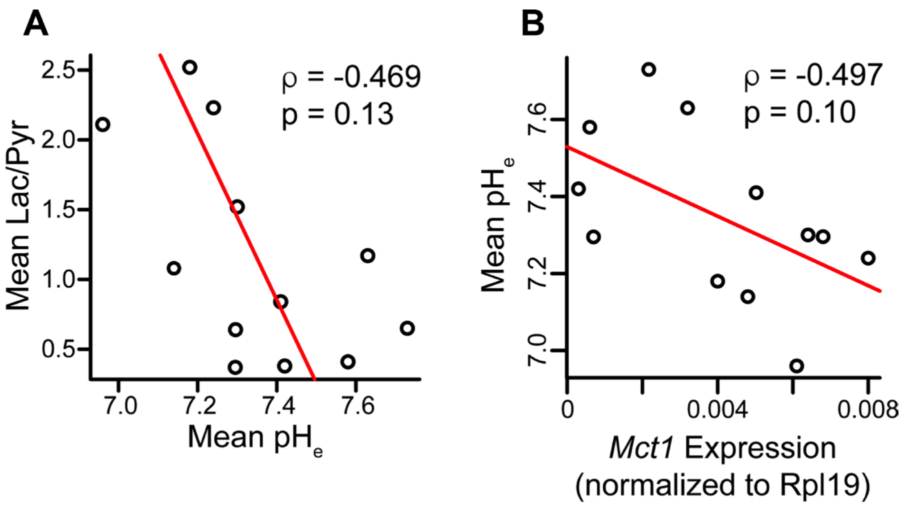

Supplementary Figure 5: Regression analyses between imaging parameters and RT-PCR data. (**A**) Spearman non-parametric regression between HP imaging metrics of mean Lac/Pyr ratio and mean pH_e was negative but not statistically significant (n = 12 lesions). (**B**) Spearman non-parametric regression between mean pH_e and *Mct1* expression was negative but not statistically significant (n = 12 lesions).

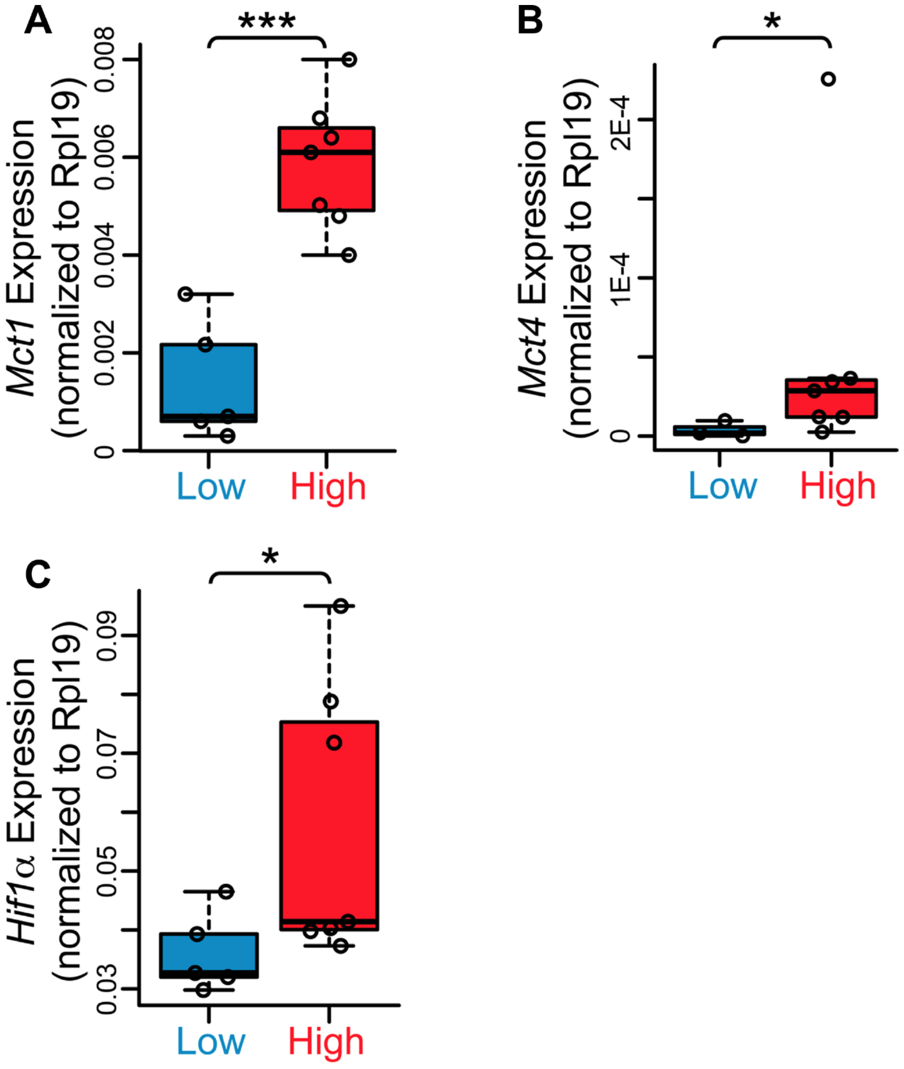

Supplementary Figure 6: Grade-dependent differences in monocarboxylate transporters and hypoxia-inducible factor. TRAMP tumors upregulate monocarboxylate transporters and hypoxia-inducible factor as they progress to an aggressive phenotype. (**A**–**C**) Scatter plots of mRNA expression for (A) *Mct1* (p = 0.00252, n = 5 low-grade, n = 7 high-grade); (B) *Mct4* (p = 0.0333, n = 3 low-grade, n = 7 high-grade); and (C) *Hif1α* genes (p = 0.0480, n = 5 low-grade, n = 7 high-grade). The *Mct4* gene expression for two low-grade lesions was below the threshold of quantification.

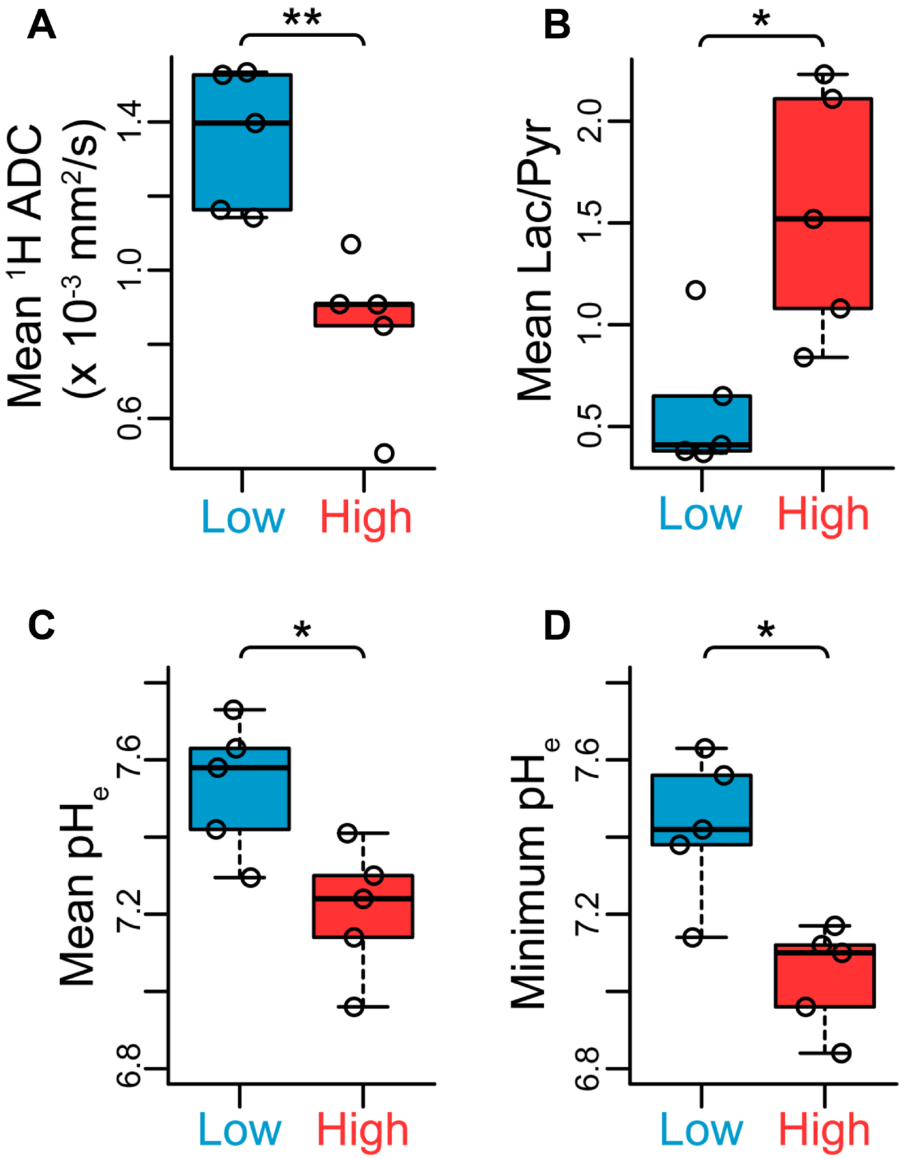

Supplementary Figure 7: Analysis of tumor ROIs including only one tumor ROI per mouse, in order to eliminate dependent samples. For each mouse with a low-grade and high-grade ROI, only the low-grade ROI was included in the analysis. (**A**) mean ^{1}H apparent diffusion coefficient (ADC), p = 0.00794; (**B**) mean Lac/Pyr ratio, p = 0.0318; (**C**) mean pH_e, p = 0.0318; (**D**) minimum voxel pH_e, p = 0.0159 (n = 5 each grade).
